# Designing in-vitro systems to simulate the in-vivo permeability of drugs

**Published:** 2014-04-08

**Authors:** Sara Cascone, Gaetano Lamberti, Giuseppe Titomanlio

**Affiliations:** Department of Industrial Engineering – University of Salerno Via Giovanni Paolo II, 132 – 84084 Fisciano (SA), Italy

**Keywords:** Permeability, Artificial membranes, Franz cell, in-vitro/in-vivo correlations, stokes radius, oral delivery

## Abstract

In this work an engineering approach, consisting in an experimental procedure and a model to derive the data, was presented and applied to improve the testing methods of pharmaceuticals. The permeability of several active molecules have been evaluated across a synthetic membrane. The permeability of these drugs measured through the artificial membrane were successfully correlated to their in-vivo permeability. The relationship with in-vivo permeability was derived, and then a rule to design systems to simulate the intestinal absorption was proposed to reduce the need for expensive and ethical problematic in-vivo measurements.

## I. INTRODUCTION

The absorption of orally administrated drugs is largely determined by their ability to cross the gastrointestinal membrane walls, quantified by the drug permeability. Most of developing drug candidates are lost because of their inappropriate pharmacokinetic properties in particular, the permeability [1]. To increase the quality of developing compounds, methods are required in the early stage of drug discovery to quantify the key factors influencing the drug absorption such as solubility and permeability, as recommended by the Biopharmaceutics Classification Scheme (BCS) [2]. A number of molecular properties which influence passive absorption of drugs have been identified: for example, octanol/water partition and distribution coefficient (log Kow), ionization state (pKa), hydrogen bond potential, and molecular size.

Alternatively, cell culture models such as Caco – 2 cell monolayer could be useful to predict gastrointestinal drug absorption. In-vitro systems based on cultured cells are strongly affected by the biological nature of the cells, for example, the apical and basolateral membranes have different lipid components, different surface charge, and different membrane – bound proteins [3]. However, this method is ra ther complex and labor intensive, and it is not applicable for high throughput.

Artificial membranes have the advantage of offering a highly reproducible and high throughput system [4]. These membranes have been compared with Caco – 2 cells and in passive diffusion studies they behave very similarly thus, for rapid prediction of transcellular absorption potential, artificial membranes are preferred because of their simplicity of use.

The aim of this work is to identify and apply a simple method which could be used to evaluate the permeability of some active molecules across a synthetic membrane and to correlate the in vitro results with the in vivo permeability.

## II. MATERIALS AND METHODS

### II.A Materials

All the materials used were provided by Sigma Aldrich (Milano, Italy). They were: propranolol hydrochlorid (CAS number 3506-09-0), diclofenac sodium (CAS number 15307-79-6), theophylline (CAS number 58-55-9), vitamin B12 (CAS number 68-19-9), and bovine serum albumin (CAS number 9048-46-8). Dissolution media were prepared using distilled water, hydrochloric acid, and sodium phosphate tribasic dodecahydrate. A cellulose acetate filter (pore size 0.2 μm) was used as filtering membrane for the permeability experiments.

## II.B Methods

**Fig 1.**
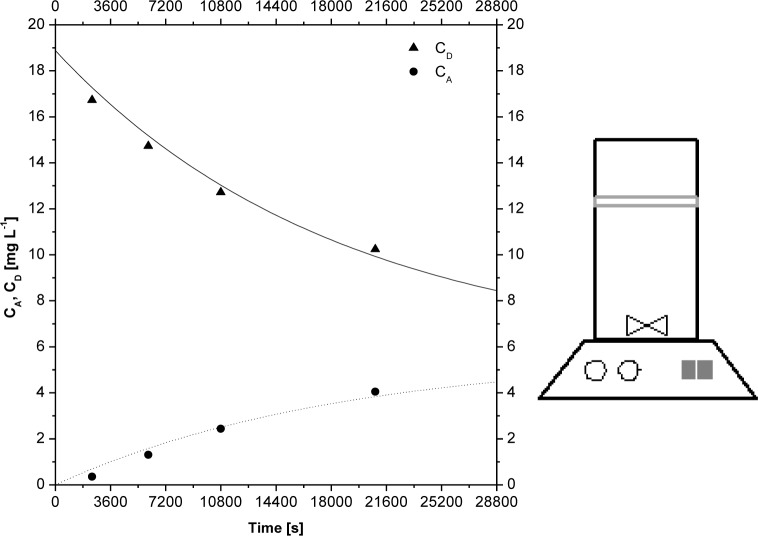
Theophylline concentration profiles both in the donor and in the acceptor compartments (on the left); Schematic representation of Franz cell (on the right).

To carry out the permeability measurement a Franz cell, which is commonly used in preclinical and in-vitro studies to evaluate the permeation of drugs, has been used. The Franz cell is characterized by the presence of two different volumes: the upper one, the donor compartment, which could be filled of a medium rich in drug content, measuring 3 ml, and the lowest one, the acceptor compartment, which could be filled of a medium poor in drug content and kept stirred, measuring 7 ml. Both the media consisted of a buffer solution at pH 6.8. The buffer solution was obtained mixing 750 ml of a distilled water/HCl solution (6.25 ml HCl of 37% wt purity) and 250 ml of a solution obtained dissolving 16 g of sodium phosphate tribasic dodecahydrate in distilled water. The volumes of the cell are separated by a membrane of area and the drug contained in the donor compartment needs to pass across the membrane to reach the acceptor compartment. All the experiments are carried on at the same pH in both of the compartments. During all the experiments, the Franz cell was immersed in a bath with controlled temperature at 37°C. To evaluate the permeability across the membrane, a buffer solution at a certain concentration of the selected active molecule is inserted in the donor compartment, while the acceptor compartment is filled of buffer solution free of drug. Then, the Franz cell is immersed in the stirred bath until a predetermined time. At the end of the run, the concentration of the active molecule is evaluated both in the donor compartment and in the acceptor one by an UV spectrometer Lambda 25 PerkinElmer. For each solution, the concentration of the molecule is evaluated at its specific absorption wavelength (λ, which is reported in table 1). The experiments are carried on for different times to obtain the concentration profile both in the donor and in the acceptor compartment (the theophylline concentration profile is shown, as example, in Fig 1).

## III. RESULTS AND DISCUSSION

The permeability of five different active molecules across an acetate cellulose membrane was evaluated. Three model active molecules (theophylline, diclofenac, and propranolol) were chosen because of their small Stokes radii and the fact that they are very well studied from the permeability point of view. Then, an active molecule with a higher molecular weight (about an order of magnitude) but a comparable Stokes radius was tested (B12) to evaluate the effect of the molecular weight on the membrane permeability. Finally, a molecule with both a Stokes radius and a molecular weight higher than the previous one was chosen (BSA) in order to evaluate the membrane permeability of a larger molecule. The last two molecules were tested only in order to evaluate the membrane features but the in vitro permeability values are not compared with the in vivo ones because the real transport mechanism of these molecules is different from the others. By the experimental set-up the concentration profiles (of the donor and the acceptor compartments) versus time were evaluated. The permeability of each molecule was then calculated solving the mass balance equations on the systems composed by the two compartments of the Franz cell. The concentration profiles versus time could be expressed by the following equations (with their initial conditions):

(1) $$\{\begin{array}{cc} \frac{d\left(c_{D}\cdot V_{D}\right)}{dt}=-J\cdot A_{s} & @t=0,c_{D}=c_{D0}\\ \frac{d\left(c_{A}\cdot V_{A}\right)}{dt}=J\cdot A_{s} & @t=0,c_{A}=c_{A0} \end{array}$$

*c_D_* and *c_A_* are the concentrations in the donor and acceptor compartment, respectively. *c_D_*_0_ and *c_A_*_0_ are the initial concentrations in the donor and acceptor compartment, respectively (initially the acceptor compartment was drug free in all the runs). *V_D_* = 3·10^−6^ m^3^ and *V_A_* = 7·10^−6^ m^3^ are the volumes of the donor and the acceptor compartment. *A_s_* = 7.85·10^−5^ m^2^ is the exchange area between the compartments. *J* is the flux across the membrane and it could be expressed as a permeability (*P*) function:

(2) $$J=P\cdot \left(c_{D}-c_{A}\right)$$

In the model equation the global fluxes have been considered in the global transport coefficient, which combines both the diffusive and the convective phenomena.

The two differential equations have to be resolved simultaneously because each of them depend on both the acceptor and the donor drug concentration. Defining δ as the difference between the donor and the acceptor drug concentration (*δ* = *c_D_* − *c_A_*), it could be obtained:

(3) $$\text{ln}\frac{\delta }{\delta_{0}}=-\frac{P}{\gamma }\cdot t$$

In which *γ* is a geometrical parameter (m^−1^):

(4) $$\gamma =\frac{1}{A_{s}}\cdot {\left(\frac{1}{V_{D}}+\frac{1}{V_{A}}\right)}^{-1}$$

**Fig 2.**
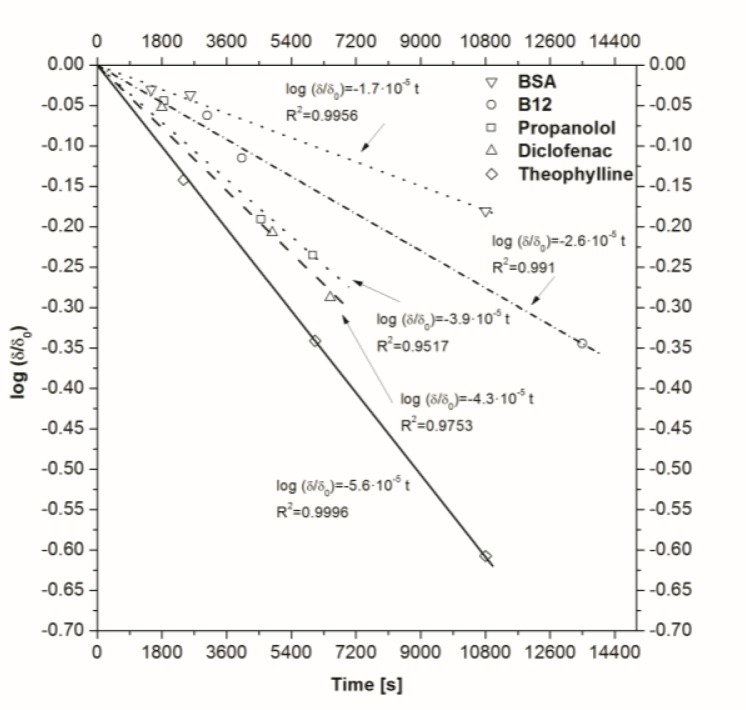
Obtained values of ln (*δ/ δ_0_*). The slope represents the ratio between the value of P and a geometrical parameter.

The obtained values of ln *(δ/* *δ_0_)* are reported in Fig 2. The slope represents the opposite of ratio between the value of *P* and *γ*. From the graph it could be seen that the most permeable molecule is theophylline and the least one is BSA.

The in-vitro permeability are reported in table 1 and compared with the in-vivo values (taken from literature).

**Fig 3.**
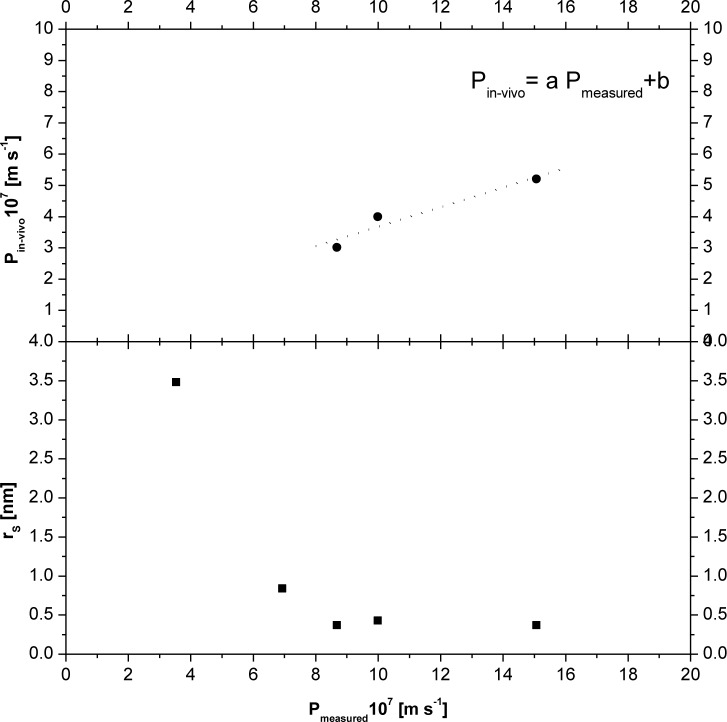
Comparison between in-vivo and measured permeability (the upper graph) and dependence of in-vitro permeability from the stokes radius of the molecules. a = 0.313; b = 0.554; R^2^ = 0.927.

In the Fig 3 (the upper graph) the in-vivo and in-vitro values of permeability are compared. It could be seen that the relation between the values is almost linear. The analyzed compounds are subjected to a passive transport across the membrane, this feature is fundamental to develop an in-vitro/in-vivo correlation. For the experimental set-up proposed in this work, a necessary condition of applicability of this relation is that the molecules analyzed have to be subjected only to passive transport across the membrane. In fact, different kind of mechanisms affects sensibly the permeability value and transport features. Therefore, by a simple method proposed in this work, it could be possible to evaluate the in-vitro permeability of some drugs and to correlate these values to the in-vivo ones. In fact, defining *J_RI_* the flux across the real intestine walls, in the real intestine the mass balance equations become:

(5) $$J_{RI}\cdot A_{RI}=A_{RI}.P_{\mathrm{in}-\mathrm{vivo}}\cdot \delta$$

In which *A_RI_* and *P_in-vivo_* are the exchange area and the permeability in the real intestine, respectively. Thus, to obtain the same mass evolution in the real intestine and in the in-vitro system, the following equation must be respected, considering that the concentration gradient between the two compartments in-vitro and between the intestine and the blood in-vivo has to be the same for each molecule to reproduce the physiological conditions:

(6) $$A_{RI}\cdot P_{\mathrm{in}-\mathrm{vivo}}\cdot \delta =A_{s}\cdot P_{\mathrm{in}-\mathrm{vivo}}\cdot \delta$$

This equation states that the two fluxes, the in-vitro one between the two Franz cell compartments and the in-vivo one between the intestinal lumen and the blood, have to be equal to guarantee the same mass exchange in the in-vitro and in-vivo conditions. Because the concentration gradient is the same, the term has to be equal in-vitro and in-vivo. Thus, to evaluate the membrane area necessary to reproduce the same transport which is realized in the real intestine, the following equation has to be used:

(7) $$A_{s}=A_{RI}\cdot \frac{P_{\mathrm{in}-\mathrm{vivo}}}{P_{\mathrm{in}-\mathrm{vitro}}}$$

Then, using the relation between in-vitro and in-vivo permeability found in this work (Fig 3), and replacing it in the equation 7, a device with the following exchange area should be built:

(8) $$A_{s}=A_{RI}\cdot \frac{a.P_{\mathrm{in}-\mathrm{vitro}}+b}{P_{\mathrm{in}-\mathrm{vitro}}}$$

As a result, it could be possible build a device with a higher exchange area than the conventional devices and, as a consequence, with an higher throughput capacity. Thus, the device can be built, in order to enhance the area and the contact surface between the donor and the acceptor fluids, for example as a concentric membrane exchanger. Moreover, to further increment the area, many devices can be placed in series.

This technique make simpler and faster the permeability evaluations. Because of the porosity of the artificial membrane is far from that of the intestinal wall, some molecules which are allowed to pass through the intestinal wall are retained on the membrane.

In Fig 3 is also reported (the lower graph) the permeability versus the Stokes radius of the molecule. It could be seen that higher the radius of the molecule, lower the permeability value. On the other hand, below a certain value of the Stokes radius (0.4 nm), the permeability value is not so strongly dependent on the molecular size.

## V. CONCLUSION

In this work a simple method to evaluate the permeability of some active molecules subjected to passive transport was pointed out. A Franz cell in which the donor and the acceptor compartments are separated by an artificial membrane was used. At predetermined times, the two concentrations were evaluated and the concentration profile could be obtained. By simple mass balance equations, also the permeability values were calculated.

In this work, experiments were carried on different active molecules. Once obtained the in-vitro permeability values, the results were compared with the in-vivo permeability and a linear correlation was found, with good agreement between the experimental results and the literature data. Moreover, the correlation between the in-vitro permeability and the Stokes radius of the molecules was analyzed founding that below a certain value of the Stokes radius, the permeability value is not so strongly dependent on the molecular size.

## Figures and Tables

**Table 1.** Physico – chemical characteristics, wavelength of UV absorption, in-vivo and in-vitro permeability of BSA, Vitamin B12, Propranolol, Diclofenac, and Theophylline.

#		Mw [g·mol^−1^]	*r_S_* [nm]	pKa	Log Kow	λ [nm]	Permeability in-vivo [m/s]	Permeability in-vitro [m/s]
1	BSA	66776	3.48 [5]	6.7 [6]	-	280	-	3.5·10^−7^
2	B12	1355.4	0.84 [7]	7.5 [8]	3.57 [9]	361	-	6.9·10^−7^
3	Propranolol	259.34	0.37 [10]	9.1 [10]	3.09 [11]	289	3.01·10^−7^ [12]	8.6·10^−7^
4	Diclofenac	296.15	0.43 [13]	4.0 [14]	1.90 [15]	275	4·10^−7^ [1]	10.0·10^−7^
5	Theophylline	180.16	0.37 [7]	8.4 [16]	−0.028 [17]	275	52·10^−8^ [18]	15.1 ·10^−7^
